# Panx1 promotes invasion-metastasis cascade in hepatocellular carcinoma

**DOI:** 10.7150/jca.32986

**Published:** 2019-09-07

**Authors:** Guangjun Shi, Chuanliang Liu, Yiming Yang, Liwei Song, Xueni Liu, Chuanxu Wang, Zhihai Peng, Hao Li, Lin Zhong

**Affiliations:** 1 Department of Hepatobiliary Surgery, The Affiliated Qingdao Municipal Hospital of Qingdao University, Qingdao, China.; 2 Department of General Surgery, The Third People's Hospital of LinYi, Linyi, China.; 3 School of Life Science, Shanghai University, Shanghai, China.; 4 Department of General Surgery, Shanghai General Hospital, Shanghai Jiao Tong University School of Medicine, Shanghai, China.

**Keywords:** Panx1, hepatocellular carcinoma, metastasis, EMT

## Abstract

**Background**: The molecular function of pannexin1 (Panx1) in different tumor types has been remained equivocal. Until now, there is no study focused on the function of panx1 in hepatocellular carcinoma (HCC). This study aimed to explore the role of Panx1 in the invasion and metastasis of HCC.

**Methods**: The expressions of Panx1 in 126 cases of HCC were analyzed by immunohistochemistry (IHC). The effects of Panx1 on HCC cell metastasis and invasion were observed by transwell. The expression levels of Panx1 and epithelial-mesenchymal transition (EMT) related proteins in HCC cells and tissues were detected by western blot and IHC. The tumor metastatic abilities were compared between Panx1 knockout mice and nude mice.

**Results**: The higher expression of Panx1 in HCC was positively correlated with tumor lymph node metastasis, TNM (tumor, node, metastasis) classification and poor prognosis (overall survival, hazard ratio [HR] 2.769, 95% confidence interval [95%CI] 1.528-5.017, *P*=0.001; disease-free survival, HR=2.344, 95%CI 1.473-3.730, *P*<0.001). Overexpression of Panx1 promoted invasion and migration of HCC cells through modulation of EMT in vitro and in vivo.

**Conclusions**: Our results suggest that the high expression of Panx1 is associated with poor HCC prognosis, providing a new clue for effective intervention for HCC metastasis.

## Introduction

Hepatocellular carcinoma (HCC) is one of the most common malignant tumors in the world; it ranked the third leading cause of cancer death 1. As it is difficult to detect HCC in early stages, only 10%-20% of patients are able to go through radical surgery 2, 3. Despite the advanced diagnosis and treatments, the prognosis of patients with HCC remains poor due to the high recurrence and metastasis rate. In addition, the mechanism of HCC is still enigmatic 4-6. Therefore, the identification of potential biomarkers and novel mechanism of metastasis are of great importance to better understand the occurrence reasons of HCC and provide potential treatments.

Panx1, a channel-forming glycoprotein, was named for its similarities with the invertebrate gap junction protein innexins 7. Panx1 is a transmembrane domain composed of four alpha helices, two extracellular loops and one carboxy terminus 8. The main function of Panx1 is to form macroporous single-membrane channels that release ATP and metabolites which play a prominent role in the exchange of information between cells 9. It is widely expressed in most mammalian cells and tissues 10. The metastasis of primary HCC was considered the main cause of cancer death 11,12. Recent studies show that Panx1 is involved in the development of many human cancers. Paul et al. reported that Panx1 channel inhibitors reduce ATP release and thereby inhibit breast cancer metastasis 13. Li et al. found that silencing Panx1 expression can reduce the proliferation of human glioma U87-MG cells 14. However, in C6 gliomas, Panx1 was reported as a tumor-suppressive factor 15. Until now, the role of Panx1 in the development of HCC has not been reported yet.

In this study, elevated expression of Panx1 in HCC was positively associated with vascular invasion, tumor metastasis and poor prognosis of patients. This study explained the related mechanism of Panx1 on the HCC invasion and metastasis, which provided a theoretical basis for the diagnosis and prognosis of HCC.

## Materials and Methods

### Patient and sample

126 pairs of HCC and adjacent noncancerous tissue were collected from the Shanghai First People's Hospital between 2009 and 2016. All patients were diagnosed as primary HCC by pathology confirmedly and did not receive any radiotherapy or chemotherapy before surgery. The fresh specimens were processed immediately after surgical removal from the patient's body, then subdivided into three equal portions for histopathologic analysis, quick frozen in liquid nitrogen, or fixated in buffered paraformaldehyde. Staging criteria of HCC were based on the TNM staging which proposed by the Union for International Cancer Control (UICC) and the American Joint Committee on Cancer (AJCC). All patients had provided written informed consent on the use of clinical specimens for biomedical research.

### Cell culture

HCC cell lines (MHCC97H, MHCC97L, MHCC-LM3, HuH7, Hepa1-6), normal liver cells (L02), and HEK 293T were purchased from the cell bank of Chinese Academy of Sciences (Shanghai, China). All cell lines were cultured in Dulbecco's minimal essential medium (DMEM, Invitrogen, NY, USA) containing 10% fetal bovine serum (FBS, Invitrogen) and 1% penicillin/streptomycin antibiotics (Invitrogen) and were incubated in an incubator with 5% CO2 at 37 °C.

### Experimental animals

C57BL/6J mice and nude mice were purchased from Shanghai Laboratory Animal Center (SLAC). Male C57BL/6 mice (specific pathogen-free (SPF)-grade) and Panx1 knockout mice (aged 6-8 weeks) were bred at the animal facility of Institute Pasteur of Shanghai under pathogen-free conditions. The nude mice were housed with free access to standard laboratory diet and water. All animal experiments in this section are approved by the Laboratory Animal Ethics Committee of the Chinese Academy of Sciences.

### Plasmids and transfection

Panx1 overexpression plasmid and Panx1 control were purchased from Shanghai Jima Pharmaceutical Technology Co. Ltd. A three-plasmid-based virus packaging cell system was established, consisting of pCMV-dR8.91, pCMV-VSVG, Panx1-OE (over-expressed) / Panx1-OE-control. The three plasmid system co-transfected into HEK 293T cells, and the virus-infected 97L and LM3 cells were collected. Puromycin 2μL was screened (concentration 10 mg/mL) after 48h. The stably expressing Panx1 of cells was verified by western blotting.

### Immunohistochemistry (IHC)

The paraffin sections were incubated with primary antibody. Each slide was designated with a score for density and intensity. The staining score and the intensity score of the positive cells on each slide were calculated using the semi-quantitative scoring method. Specifically, staining scores were assigned according to the percentage of positive tumor cells, as follows: 1 (up to 25% positive cells), 2 (25%-50% positive cells), 3 (50%-75% of positive cells), and 4 (more than 75% positive cells). The intensity scores were set between 0 and 3, which are designated as follows: 0, no staining; 1, weak staining; 2, moderate staining; and 3, strong staining. The final results ranging from 0 to 12 were obtained with the multiple of the staining score and the intensity score. A total score of 0-6 indicates low expression, whereas a total score of 7-12 indicates high expression.

### Cell migration and invasion assays

The invasive abilities of 97L and LM3 cells were detected by trans-well culture system. For invasion, the trans-well membrane of the upper chamber coated with Matrigel (BD Biosciences Discovery Labware, Woburn, MA, USA) was used. The chambers were rehydrated with serum-free medium for 2 h at 37℃ in an incubator. The top chambers were subsequently hydrated with 200 ul of cell suspension (containing 1 × 105 cells). The bottom chambers were added with 500ul of medium containing 10% FBS as a chemoattractant. After culturing for 24 hours at 37℃, the transmembrane cells on the lower surface of the membrane were fixed with formaldehyde at room temperature for 5 min, stained with crystal violet for 20 min, washed three times with clean water, and counted under a microscope.

### Western blot analysis

Western blot analysis was implemented on standard procedures 16. The following antibodies were used in the experiments: anti-GAPDH antibody (Abcam, Cambridge, UK), β-actin antibody (Abcam, Cambridge, UK), anti-Panx1 antibody (Abcam, Cambridge, UK), anti-E-cadherin antibody (Cell Signaling Technology, BO, USA), anti-Vimentin antibody (Cell Signaling Technology, BO, USA), anti-Snail antibody (Cell Signaling Technology, BO, USA), anti-MMP2 antibody (Cell Signaling Technology, BO, USA), total AKT antibody (Cell Signaling Technology, BO, USA), and p-AKT (Ser 473) (Cell Signaling Technology, BO, USA). Cell and tissue sample lysates were electrophoresed using 10% sodium dodecyl sulfate-polyacrylamide gels and then transferred onto polyvinylidene difluoride membranes (Millipore, USA). The membranes were incubated with primary antibodies for 1-2 h at room temperature overnight at 4 °C and then incubated with the related secondary antibody for 1h at room temperature. Protein detection was performed using an enhanced chemiluminescence kit (Ab Frontier), and the blots were exposed to X-ray films. Band intensities were quantified using ImageJ software (version 1.43; National Institutes of Health, Bethesda, MD, USA) with GAPDH as the loading control.

### In vivo detection of tumor invasion and metastasis

Ten nude mice were randomly divided into two groups. LM3 cells (1 × 10^6^ cells/mice) stably transfected with retrovirus-Panx1 or retrovirus-control vector were injected by tail vein injection. The mice were sacrificed by dislocation before observing the lung metastasis two weeks later. The tumor metastases were compared between Panx1 knockout mice and wild-type mice. Hepa1-6 cells were planted in the armpit of mice and the tumor size was measured regularly.

### Statistical Analysis

Calculations were performed with GraphPad InStat, Version 5.0 (GraphPad Prism Software, San Diego, CA, USA). Student's two-tailed t-test was utilized for all data analysis and values were expressed as the mean ± standard error of the mean acquired from at least two independent experiments. *P*<0.05 was considered to indicate a statistically significant difference.

## Results

### High Panx1 expression was an independent prognostic factor for patients with resectable HCC

**Figure 1:**
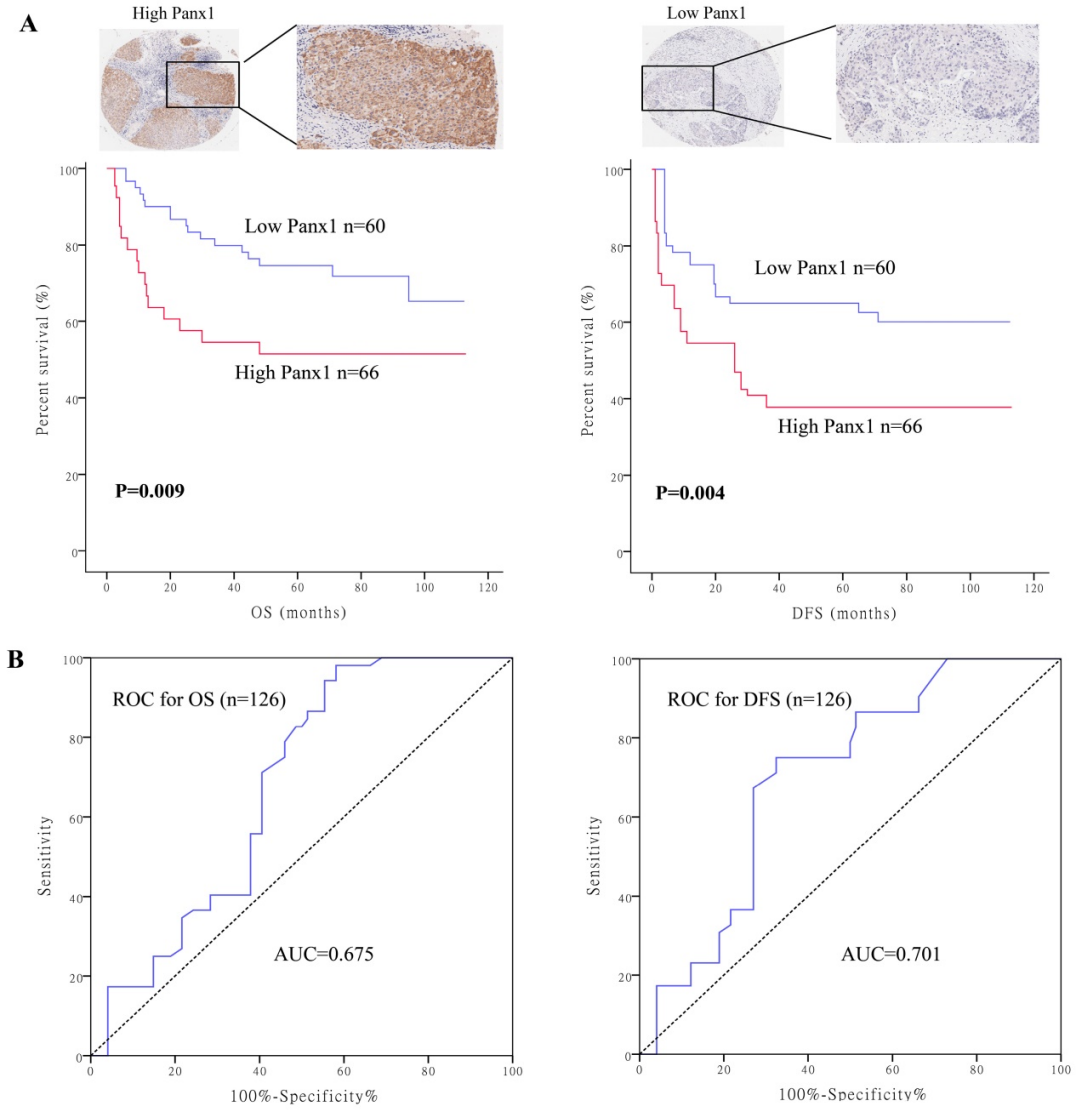
** High Panx1 expression was an independent prognostic factor for patients with resectable HCC**. (A) Kaplan-Meier showed that patients with high Panx1 expression in tumors displayed short overall survival (*P*=0.009) and disease-free survival (*P*=0.004) compared with those showing low Panx1 expression. (B) ROC curve of Panx1 for OS (AUC=0.675) and DFS (AUC=0.701) in PDAC patients.

To determine the clinical significance of Panx1, we performed survival analysis in 126 patients with HCC. Baseline characteristics of the patients are listed in Supplementary table 1. Patients were divided into two groups based on their Panx1 expression levels. Panx1 expression levels was positively correlated with microvascular involvement (*P*=0.008) and tumor lymph node metastasis stages (*P*=0.020) in patients with HCC. Patients with high Panx1 expression in tumors displayed a more advanced disease state (stages 3 and 4) than patients with low expression of Panx1 in tumors (*P*=0.022). Kaplan-Meier analysis showed that patients with high Panx1 expression in HCC tissues displayed shorter overall survival (OS) and disease-free survival (DFS) than those in patients with low Panx1 expression (*P*= 0.009 and *P*= 0.004, respectively) (Figure 1A). A receiver operating characteristic (ROC) curve analysis were performed to evaluate the prognosis value of Panx1 and the results showed that the area under the curve (AUC) for Panx1 expression associated with OS was 0.675 and DFS 0.701, respectively (Figure 1B). The Cox regression analysis displayed that Panx1 expression (HR=2.344, *P*<0.001), Microvascular involvement (HR=3.426,* P*=0.005), and TNM stage (HR=4.029, *P*=0.007) were significantly associated with DFS in HCC patients (Table 1). Panx1 expression (HR=2.769, *P*=0.001), and TNM stage (HR=10.233, *P*=0.032) were significantly associated with OS in HCC patients (Table 2). These findings suggest that Panx1 expression was considered independent prognostic factors for HCC.

### Panx1 promoted the invasion and metastasis of HCC cells in vitro and in vivo

**Figure 2:**
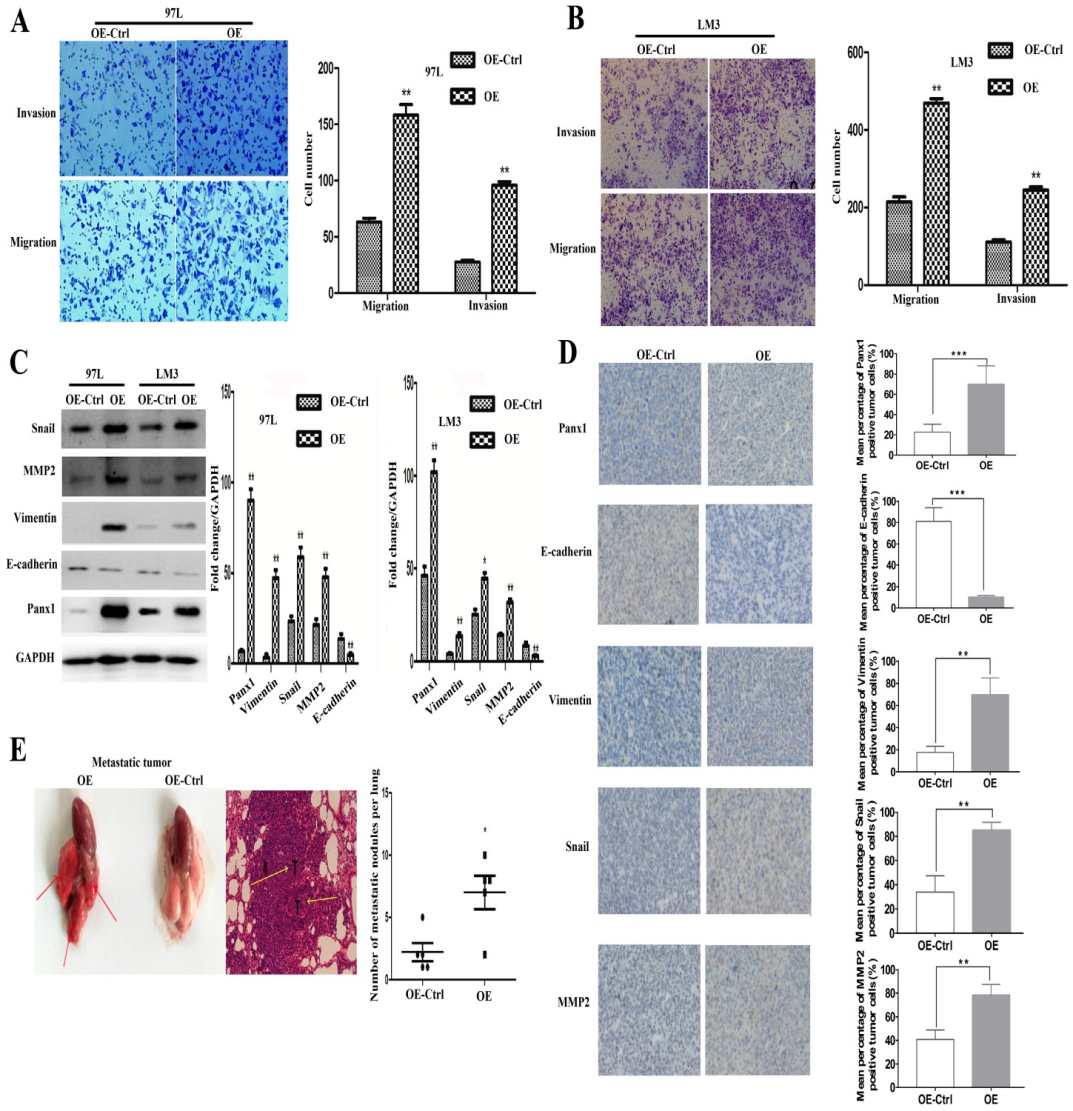
** Overexpression of Panx1 promoted cell invasion and metastasis in vitro and in nude mice.** (A, B) Transwell invasion and metastasis assay showed that overexpression of Panx1 enhanced the invasion and metastasis ability of HCC cell lines (97L cells and LM3 cells). (C) Western blot detected the expression of Panx1, E-cadherin, Vimentin, MMP-2, and Snail in 97L and LM3 cells transfected with OE or OE-Ctrl. (D) IHC showed that the expression of Vimentin, Snail, and MMP2 protein increased in tumors formed from the Panx1-transfected HCC cells than that in control cells, while E-cadherin protein expression was reduced in the Panx1 overexpression group. (E) Overexpression of Panx1 enhanced lung metastasis of HCC in nude mice.

LM3 and 97L cells were transfected with the retrovirus-Panx1 or retrovirus-control vector. Trans-well results showed that Panx1 overexpression significantly promoted the invasion and metastasis of LM3 and 97L cells (Figure 2A, B). Several EMT-related genes, the expressions of Snai1, Vimentin and MMP2 were up-regulated whereas E-cadherin was down-regulated by overexpression in HCC cells (Figure 2C). In subcutaneous tumor in nude mice, IHC showed that the Vimentin, Snail, and MMP2 positive staining were significantly more frequent in tumors of Panx1-transfected HCC cells than that in control cells, while E-cadherin protein expression was reduced in the Panx1 overexpression group, indicating that overexpression of Panx1 was more helpful to the occurrence of EMT (Figure 2D). To testify whether Panx1 overexpression contributes to tumor metastasis in vivo, we injected LM3-stable cells (overexpression and control) into the tail vein of nude mice. These results indicated that Panx1 overexpression significantly promoted lung metastasis in nude mice (Figure 2E).

### Panx1 promoted EMT of HCC were dependent on AKT signaling

**Figure 3:**
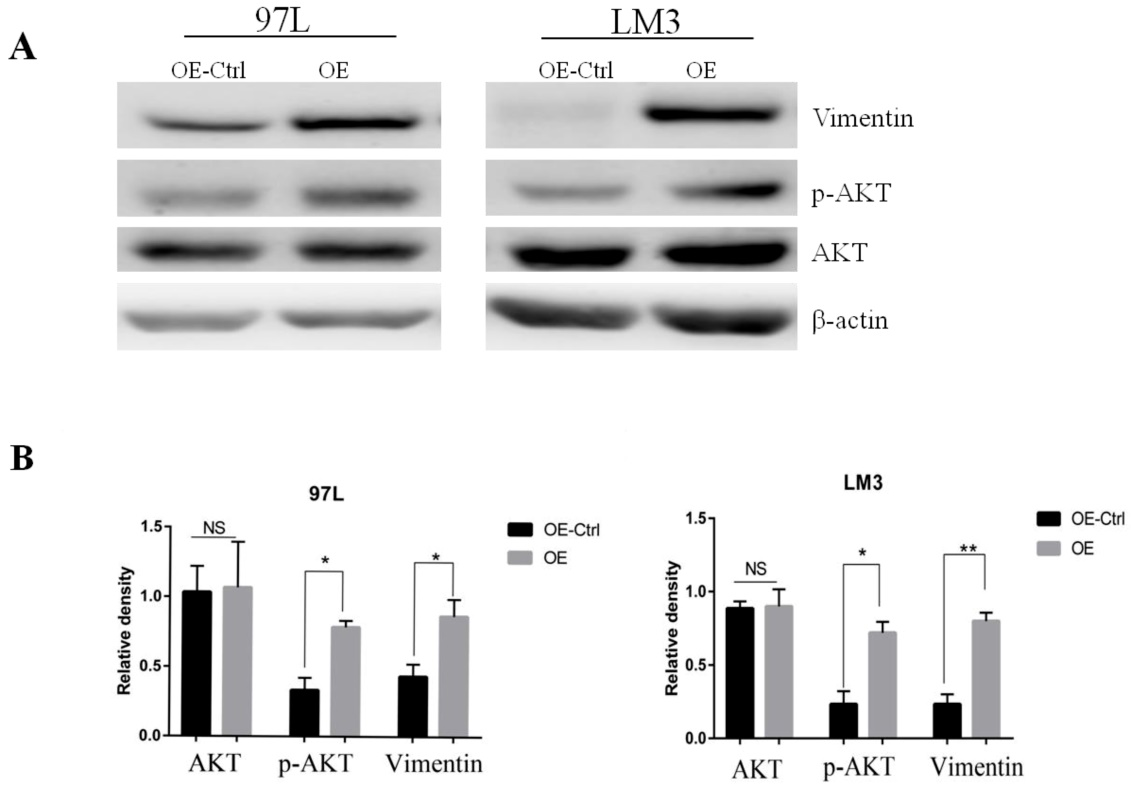
** Panx1 promoted EMT of HCC cells by AKT phosphorylation.** (A) Western blot detected the expression of AKT, p-AKT, and Vimentin in 97L and LM3 cells transfected with OE or OE-Ctrl. (B) The average relative density of AKT, p-AKT, and Vimentin in 97L and LM3 cells.

To search for the potential link between Panx1 and the EMT signaling, we examined the molecular alterations in AKT signaling pathways that were important in EMT induction. The results showed that Panx1 promoted EMT of HCC via phosphorylated AKT in both LM3 and 97L cells (Figure 3).

### Panx1 knockout inhibited metastasis in vivo

**Figure 4:**
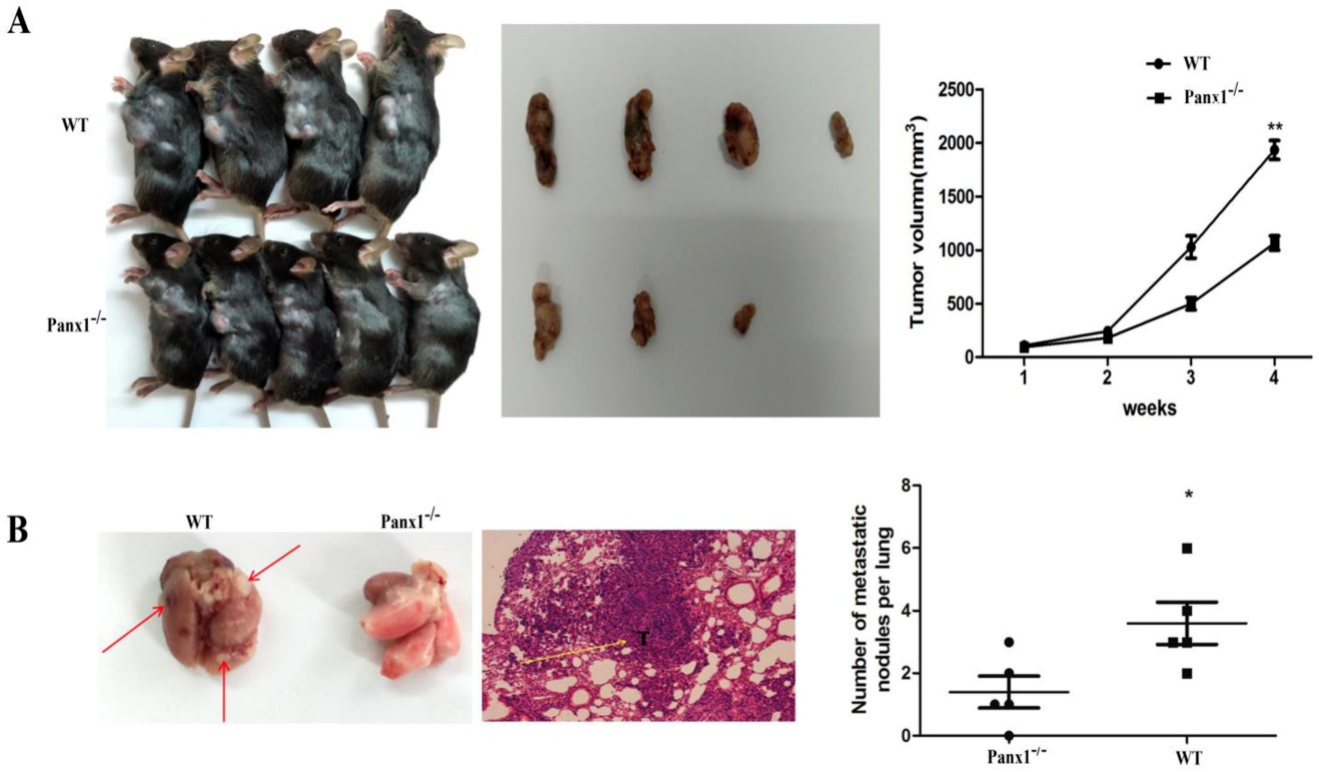
** Deleted of Panx1 suppressed tumor cells lung metastasis in Panx1 knockout mice.** (A) Panx1 knockout mice suppressed HCC growth. (B) Panx1 knockout mice suppressed lung metastasis of HCC.

The hepa1-6 cells were injected in the axilla of Panx1 knockout mice and wild-type (WT) mice respectively. The tumor volume in Panx1 knockout mice was significantly smaller than those in WT mice (Figure 4A). The number of lung metastasis tumor nodules in Panx1 knockout mice was significantly reduced than that in WT mice (Figure 4B). These data indicated that Panx1 knockout inhibited its metastasis.

## Discussion

Although the clinical diagnosis and surgical treatment of HCC have made great progress, the mortality rate of HCC is still amongst the top three highest. The majority of patients with HCC have undergone intrahepatic or even distal metastasis in clinical treatments. Therefore, the development of new hepatocellular carcinoma diagnosis and prognostic factors is of urgent significance. Previous studies have revealed that Panx1 channels are involved in the regulation of cellular functions mainly through the mediation of ATP release and paracrine signaling 17-19. However, this study mainly focused on the relationship between the expression level of Panx1 and the clinical indications in HCC patients as well as the role of Panx1 in HCC metastasis.

The expression of Panx1 was positively correlated with tumor TNM staging and venous invasion, negatively correlated with overall survival. At the same time, Panx1 expression levels and venous invasion could be used as an independent prognostic factor for the patients with HCC. Consistent with this, Stewart et al. reported that high expression levels of Panx1 are associated with poor OS of breast cancer patients 20. This provides a new direction for us to find a novel indicator of HCC prognosis.

As we know metastasis is the main factor for HCC patients with a poor prognosis, and we speculated that Panx1 may have an related impact on the biological function of hepatocellular carcinoma cells. We established a Panx1 over-expressing hepatocellular carcinoma cell line and found that Panx1 overexpression promotes cell migration and invasion. Consistent with this, Silvia reported that the loss of Panx1 expression inhibits cell proliferation and migration on melanoma 21. In Lai's report, on the contrary, they found that overexpression of Panx1 in gliomas suppresses cell malignancy 15. The above researches on Panx1 in different tumors indicate that Panx1 plays a complex role in the development of cancer, resulting in different data which might relate to the genetic background of tumor cells, cell culture conditions or analytical methods of the researchers, which needs more researchers to explore and discuss. In this study, Panx1 was found to promote the transmembrane transformation of hepatocellular carcinoma cells by promoting the expression of Snai1, an EMT-related transcription factor. The expression of E-cadherin protein was down-regulated, the expression of vimentin and snai1 protein was up-regulated, and the expression of MMP2 protein was up-regulated, suggesting that Panx1 overexpression may promote cell migration and invasion. Subsequently, we used trans-well to detect the metastatic potential of the cells, comparing it with the control group, the ability of migration and invasion of the hepatocarcinoma cells with Panx1 overexpression group was significantly increased.

This showed that Panx1 can regulate cell migration and invasion by promoting epithelial-mesenchymal transition. However, the main focus of our follow-up study is to find out how Panx1 affects and regulates EMT-related gene expression. To further clarify the role of Panx1 in tumorigenesis, we established a tumorigenic model of subcutaneous nude mice with HCC cells and found that Panx1 promoted the tumor volume significantly. Meantime, we also planted hepa1-6 cells in the axilla of Panx1 knockout mice and obtained the same results, the lung metastasis of mice in the Panx1 knockout group was significantly lower than the wild-type. This showed that Panx1 can promote cell migration, which is consistent with previous experiments in vitro.

This present study investigated the clinical significance of Panx1 in HCC patients, but it still has some limitations. First, although high expression of Panx1 can predict the poor prognosis in HCC, it still lacks of registered clinical trial. Second, although we confirmed Panx1 promotes HCC invasion and metastasis in vitro and in vivo (included nude mice and Panx1 knockout mice), as well as EMT-related expression, we did not provide the detailed mechanism about how Panx1 promotes EMT-related expression.

The above studies showed that Panx1 is involved in the development of HCC and promote cell migration. In summary, this study firstly proved the role of Panx1 in the development of HCC, however, the mechanism remains to be determined.

## Supplementary Material

Supplementary table.

## Figures and Tables

**Table 1:** Univariate and multivariate analysis of factors associated with DFS of HCC patients

Variables	Hazard ratio (95% CI)	*P* value
Univariate analysis		
Panx1 (low, moderate vs high)	1.824 (1.187-2.801)	0.006
Gender (male vs female)	1.446 (0.342-6.112)	0.616
Age (>50 vs ≤50)	1.188 (0.534-2.647)	0.673
HBV (positive vs negative)	0.383 (0.051-2.854)	0.349
Tumor size (>5cm vs ≤5cm)	3.082 (1.423-6.674)	0.004
Liver cirrhosis (yes vs no)	0.544 (0.219-1.351)	0.189
Microvascular involvement (positive vs negative)	4.803 (2.223-10.380)	<0.001
Differentiation (Poorly vs well+moderately)	2.721 (1.088-6.805)	0.032
TNM stage (III vs I-II)	5.144 (1.938-13.655)	0.001
Lymph node metastasis (yes vs no)	1.494 (0.563-4.729)	0.102
AFP (>20ng/ml vs ≤20ng/m)	0.893 (0.360-2.212)	0.806
Multivariate analysis		
Panx1 (low, moderate vs high)	2.344 (1.473-3.730)	<0.001
TNM stage (III vs I-II)	4.029 (1.476-10.999)	0.007
Microvascular involvement (positive vs negative)	3.426(1.451-8.090)	0.005

P<0.05 was considered statistically significant

**Table 2:** Univariate and multivariate analysis of factors associated with OS of HCC patients

Variables	Hazard ratio (95% CI)	*P* value
Univariate analysis		
Panx1 (low, moderate vs high)	3.064 (1.693-5.544)	<0.001
Gender (male vs female)	0.867 (0.199-3.771)	0.849
Age (>50 vs ≤50)	1.118 (0.419-2.979)	0.824
HBV (positive vs negative)	0.764 (0.625-1.201)	0.232
Tumor size (>5cm vs ≤5cm)	2.265 (0.892-5.750)	0.085
Liver cirrhosis (yes vs no)	0.333 (0.124-0.892)	0.029
Microvascular involvement (positive vs negative)	7.261 (2.782-18.951)	<0.001
Differentiation (Poorly vs well+moderately)	1.663 (0.481-5.756)	0.422
TNM stage (III vs I-II)	18.908 (2.509-142.487)	0.004
Lymph node metastasis (yes vs no)	2.854 (1.051-7.972)	0.047
AFP (>20ng/ml vs ≤20ng/m)	1.508 (0.566-4.019)	0.412
Multivariate analysis		
Panx1 (low, moderate vs high)	2.769 (1.528-5.017)	0.001
TNM stage (III vs I-II)	10.233 (1.226-85.410)	0.032

*P*<0.05 was considered statistically significant
