# Supplementary material for: Panx1 promotes invasion-metastasis cascade in hepatocellular carcinoma
Source: J Cancer. 2019 Sep 7;10(23):5681–8. doi: 10.7150/jca.32986 (PMC6843873; doi:10.7150/jca.32986)
Supplement: Supplementary file 1 — Supplementary table. [file jcav10p5681s1.pdf]

Supplementary table 1 Clinicopathological features and the correlations of Panx1 expressions with these factors in patients with HCC

Parameters	High expression (n=66)	Low expression (n=60)	*p* value
Gender			0.488
Male	53	51	
Female	13	9	
Age			0.292
<50	37	28	
⩾50	29	32	
HBV			0.844
Postive	44	39	
Negative	22	21	
Tumor size			0269
>5cm	34	25	
⩽5cm	32	35	
Cirrhosis			0.209
Yes	30	34	
No	36	26	
Microvascular involvement			0.008
Positive	19	6	
Negative	47	54	
TNM stage			0.022
III	17	6	
Ⅰ and Ⅱ	49	54	
Lymph node metastasis			0.020
Yes	23	10	
No	43	50	
AFP			0.312
Negative (⩽20ng/ml)	40	31	
Positive (>20ng/ml)	26	29	
